# Genome-Wide Association Studies in Hepatocellular Carcinoma: Aetiology-Specific Susceptibility, Functional Interpretation, and Clinical Translation

**DOI:** 10.3390/genes17070759

**Published:** 2026-06-30

**Authors:** Siwei Zhang, Xiaohang Long

**Affiliations:** 1 Center for Applied Bioinformatics, St. Jude Children’s Research Hospital, Memphis, TN 38105, USA; siwei.zhang@stjude.org; 2 School of Biomedical Sciences, The Chinese University of Hong Kong, Sha Tin, Hong Kong SAR, China

**Keywords:** hepatocellular carcinoma, HCC, genome-wide association study, genetic predisposition to disease, genetic risk score, polymorphism, single nucleotide, molecular epidemiology

## Abstract

Background/Objectives: Hepatocellular carcinoma (HCC) arises through heterogeneous pathways involving chronic hepatitis B virus infection, hepatitis C virus infection, alcohol-related liver disease, metabolic dysfunction-associated steatotic liver disease, fibrosis, cirrhosis, and environmental exposures. Genome-wide association studies (GWASs) have identified host germline loci associated with HCC susceptibility, but interpretation is complicated by aetiology, ancestry, liver disease stage, and the definition of controls. This narrative review examines current GWAS evidence for HCC, with emphasis on aetiology-specific susceptibility, functional interpretation, cross-disorder genetic effects, and clinical translation. Methods: Studies were identified through iterative searches of PubMed/PMC, publisher pages, academic search tools, and citation tracking, supplemented by targeted searches for major HCC-associated loci. Sources were chosen based on relevance to GWAS discovery, replication, meta-analysis, functional interpretation, polygenic risk modelling, or HCC risk stratification, rather than by a formal systematic review protocol. Results: Viral HCC studies most often implicate immune regulation and antigen presentation, including *MICA*, *HLA-DQ*, *HLA-DQB1*, HLA class I, *HCP5*, *STAT4*, *DEPDC5*, and *FAM114A1*. Alcohol-related, metabolic, and non-viral HCC studies more often implicate hepatic lipid metabolism, telomere biology, iron metabolism, steatosis, and cirrhosis-related pathways, including *PNPLA3*, *TM6SF2*, *TERT*, *HSD17B13*, *APOE*, *HFE*, and *MTARC1*. Recent studies increasingly combine GWASs with fine-mapping, functional annotation, transcriptomic analyses, and risk modelling. Conclusions: HCC genetic susceptibility is highly aetiology-specific and overlaps with other liver and metabolic disorders, but discoveries from genetic studies have not yet been translated into routine clinical practice. Future work should prioritise multi-ancestry cohorts, disease-stage-aware controls, functional validation, and prospectively tested genetic risk models.

## 1. Introduction

Hepatocellular carcinoma (HCC) is the principal histological form of primary liver cancer and remains a major contributor to cancer mortality worldwide. Liver cancer accounted for approximately 866,136 new cases and 758,725 deaths globally in 2022, indicating the continuing need for better prevention, surveillance, and risk stratification [1]. HCC usually develops in the setting of chronic liver injury, particularly cirrhosis, chronic HBV infection, chronic HCV infection, alcohol-related liver disease, and MASLD or MASH, with the latter representing the current terms corresponding broadly to historical NAFLD and NASH categories [2,3,4]. Although these clinical and environmental risk factors are well established, only a subset of exposed individuals develops HCC, suggesting that inherited host factors modify the transition from chronic liver disease to malignancy.

It should be noted that the research on steatotic liver disease (SLD) reflects its evolving nomenclature. In 2023, a multisociety Delphi consensus recommended SLD as the umbrella term for hepatic steatosis, with MASLD replacing non-alcoholic fatty liver disease (NAFLD) and MASH replacing non-alcoholic steatohepatitis (NASH) [3,4]. In this review, MASLD and MASH are used for current terminology, whereas NAFLD and NASH are retained when referring to study names, historical diagnostic categories, or source articles that used the older nomenclature.

Genome-wide association studies (GWASs) provide an unbiased approach for identifying common germline variants associated with disease susceptibility. In HCC, GWASs have been used to compare genotype frequencies between cases and controls across distinct aetiological settings, including chronic HBV carriers, chronic HCV carriers, post-sustained virological response (SVR) cohorts, alcohol-related cirrhosis, non-viral HCC, and MASLD-associated liver disease. Together, these studies indicate that HCC is not a single genetic phenotype. Inherited risk appears to be shaped by interactions among viral exposure, immune regulation, hepatic fat handling, fibrosis biology, telomere regulation, metabolic state, ancestry, and environmental exposures.

This review explores genome-wide association studies (GWASs) for HCC, focusing on specific aetiological loci, methodological rigour, functional implications, and potential clinical applications. Given that the existing literature predominantly addresses HCC rather than cholangiocarcinoma or mixed primary liver cancers, we use the term “hepatocellular carcinoma” here primarily to refer to HCC. Rather than presenting a systematic review, this narrative synthesis prioritises peer-reviewed primary GWAS research, replication studies, meta-analyses, and integrative genetic investigations.

## 2. Review Scope and Source Identification

Research on GWASs in HCC has evolved through several interconnected phases. The initial focus was on studies of the aetiology of chronic viral hepatitis. This was particularly prominent in cohorts affected by HBV-related HCC in China and HCV-related HCC in Japan. These studies successfully identified various susceptibility signals, including *KIF1B*, *MICA*, *DEPDC5*, *STAT4*, and *HLA-DQ* [5,6,7,8]. A second phase addressed replication, ancestry transferability, and immune-region refinement, including HLA class I and *HCP5* analyses that clarified the role of antigen presentation and major histocompatibility complex variation in viral hepatocarcinogenesis [9,10,11,12]. More recent studies have extended the field towards alcohol-related, MASLD-related, post-SVR, and non-viral HCC, while incorporating meta-analysis, TWASs, co-localisation, Mendelian randomisation, PRS, and functional assays to move from locus discovery towards functional prioritisation, cautious causal-hypothesis generation, and clinical translation [13,14,15,16,17,18,19,20,21]. However, it is worth noting that HCC-related GWASs are still limited when compared with the extensively studied cancer/tumour types, such as breast, ovarian, and prostate cancers [22].

This article was developed as a narrative review rather than a systematic review. Relevant peer-reviewed studies of germline genetic susceptibility to HCC were identified through iterative searches of PubMed/PMC, publisher pages, academic search tools, citation tracking from key papers, and targeted locus searches. Search phrases included “GWAS hepatocellular carcinoma susceptibility”, “hepatocellular carcinoma genome-wide association study”, “HBV-related hepatocellular carcinoma GWAS”, “HCV-related hepatocellular carcinoma GWAS”, “alcohol related hepatocellular carcinoma GWAS”, “MASLD, MASH, NAFLD, and NASH hepatocellular carcinoma GWAS *PNPLA3*”, and “polygenic risk score hepatocellular carcinoma GWAS”. Targeted searches were also conducted for major loci discussed in the review, including *KIF1B*, *MICA*, *DEPDC5*, *STAT4*, *HLA-DQ*, HLA-DP, *TERT*, *PNPLA3*, *TM6SF2*, *FAM114A1*, *PWRN4*, and *HSD17B13* (Supplementary File S1).

Sources were chosen purposively for their relevance to the narrative themes of this review, including GWAS discovery, independent replication, meta-analysis, phenotype definition, functional follow-up, TWASs, Mendelian randomisation, PRS development, and clinical risk stratification. The search was not intended to produce an exhaustive systematic evidence map, and no formal PRISMA-style screening workflow, search-date protocol, or count of included/excluded studies were applied. Studies concerned only with somatic tumour mutations or expression-only bioinformatics analyses without evidence of germline association were used only when they helped interpret the biological context, rather than as core GWAS evidence.

## 3. Methodological Considerations in HCC GWASs

The design of an HCC GWAS plays a crucial role in how its findings are interpreted. When we compare HCC cases with healthy population controls, we may uncover variants linked not just to HCC but also to chronic liver disease, viral infections, fibrosis, or even a mix of these pathways. On the other hand, comparing HCC cases with chronic HBV or HCV carriers who do not have HCC offers more direct insights into the journey from infection to cancer. Likewise, using cirrhosis-matched controls is more effective for identifying variants related to malignant transformation in high-risk patients, while post-SVR controls help address the ongoing risk following HCV treatment.

Population stratification is another central issue. Early HBV and HCV GWASs were largely conducted in East Asian populations, reflecting regional disease burden and cohort availability [5,6,7,8,9]. More recent studies in Europe and North America have expanded the literature towards alcohol-related, MASLD-associated, and non-viral HCC [13,14,15,16]. However, many ancestry groups remain underrepresented, and PRS portability is likely to be limited when discovery cohorts and target populations differ in allele frequency, linkage disequilibrium, environmental exposure, and baseline disease prevalence.

Most signals identified in genome-wide association studies (GWASs) do not automatically imply causation. The associated variants often reside in noncoding regions or merely represent broader blocks of linkage disequilibrium. As a result, making stronger inferences requires methods such as fine-mapping, expression quantitative trait locus (eQTL) analysis, co-localisation studies, transcriptome-wide association studies (TWASs), Mendelian randomisation, cell-type-specific functional annotation, and experimental validation. While the most robust studies on HCC effectively integrate statistical associations with biological evidence, numerous loci still lack a clear mechanistic understanding.

Several general limitations of GWASs should temper interpretation of HCC susceptibility loci. Discovery-stage effect estimates may be inflated by winner’s curse and small-study effects, particularly when modest sample sizes are subdivided by aetiology, ancestry, viral status, or cirrhosis stage. Common-variant arrays also explain only part of inherited risk; rare variants, structural variants, haplotype effects within the HLA region, gene–environment interactions, and ancestry-specific linkage disequilibrium may contribute to missing heritability. These limitations are especially relevant in HCC because exposures such as HBV or HCV infection, alcohol use, MASLD, aflatoxin, antiviral treatment, and fibrosis stage can modify genetic effects. Therefore, top GWAS loci should be interpreted as prioritised statistical signals that require replication, fine-mapping, exposure-aware modelling, and functional validation before they are used to support mechanistic or clinical claims.

## 4. Current Status of GWASs in HCC

The GWAS literature in HCC is best understood as a set of aetiology-specific discoveries rather than a single unified susceptibility map. Early HCC GWAS research in East Asian populations was dominated by viral hepatitis-associated disease, particularly HBV-related HCC in Chinese cohorts, with parallel studies of HCV-related HCC in Japanese cohorts also contributing important immune-region findings [5,6,7,8,9]. More recent work has expanded towards alcohol-related, MASLD-related, post-SVR, and non-viral HCC in European, North American, and multi-ancestry cohorts. Across these settings, the genetic architecture differs by aetiology, as detailed below.

### 4.1. Viral HCC

HBV-related HCC has been a major focus of early GWAS research. Zhang et al. identified 1p36.22 as a susceptibility locus in chronic HBV carriers of Chinese ancestry, with rs17401966 in the *KIF1B* region showing strong statistical association after discovery and replication across independent samples [5]. This study remains foundational because it established the feasibility of GWAS discovery in HBV-related HCC. However, subsequent replication studies and meta-analyses have shown that the *KIF1B* association is weaker and less consistently reproduced than several immune-region signals, suggesting that ancestry, control definition, linkage disequilibrium structure, or regional genetic heterogeneity may influence its reproducibility [10,11]. By contrast, HLA-related loci, including *HLA-DQ*, HLA class I, *HLA-DQA1*/*HLA-DRB1*, and related major histocompatibility complex signals, have emerged as some of the strongest and most reproducible findings in viral-associated HCC, consistent with the central role of antigen presentation and host immune response in hepatitis-associated hepatocarcinogenesis [6,9,12,23].

More consistently replicated HBV-related findings implicate immune signalling and antigen presentation. Jiang et al. identified variants in *STAT4* and *HLA-DQ* associated with HBV-related HCC in a large Chinese GWAS followed by validation in multiple cohorts [6]. The *STAT4* locus is biologically plausible because *STAT4* participates in cytokine signalling and immune response, while *HLA-DQ* points towards antigen presentation and adaptive immunity. HLA-focussed studies have further refined the immune-region signal: Sawai et al. identified susceptible genetic variants in the HLA class I region for HBV-related HCC, using a Japanese GWAS with replication in Japanese, Hong Kong Chinese, and Thai cohorts [12].

HCV-related HCC GWASs also point towards immune regulation. Kumar et al. identified *MICA* rs2596542 as a susceptibility locus for HCV-induced HCC in a Japanese GWAS with replication [7]. The risk allele was associated with lower soluble MICA levels, a finding consistent with the hypothesis of impaired immune surveillance rather than providing proof of a confirmed mechanism [7]. Miki et al. identified variation in *DEPDC5* associated with progression to HCC in chronic HCV carriers [8], and subsequent meta-analytic work has evaluated *DEPDC5* polymorphisms as cumulative evidence has expanded [24]. European HCV-associated HCC studies further implicated the major histocompatibility complex through *HCP5*, supporting the view that inherited variation in immune-region genes influences the carcinogenic consequences of chronic viral hepatitis [9].

The clinical landscape of HCV-related HCC has significantly evolved due to the widespread adoption of direct-acting antiviral therapy. While viral eradication lowers the risk of HCC, it does not fully eliminate it, especially in patients with advanced fibrosis or cirrhosis. Suda et al. conducted a GWAS on HCC following HCV eradication and identified *PWRN4* as a risk locus in the post-SVR setting [17]. This study addresses residual carcinogenic risk after viral cure and may eventually support individualised post-SVR surveillance.

### 4.2. Alcohol-Related, MASLD-Related, and Non-Viral HCC

As the epidemiology of liver disease shifts, GWAS research now addresses alcohol-related, MASLD-related, and non-viral HCC. In these settings, the most reproducible loci often involve hepatic lipid metabolism, hepatocyte injury, telomere biology, and metabolic stress rather than classical viral immune responses. *PNPLA3* rs738409 and *TM6SF2* rs58542926 are among the most consistently implicated variants. Yang et al. evaluated *PNPLA3* and *TM6SF2* across aetiologies and reported associations with HCC risk, particularly in alcohol-related liver disease. In that study, *PNPLA3* rs738409 showed a strong association with alcohol-related HCC, and a genetic score incorporating minor alleles of *PNPLA3* and *TM6SF2* showed a dose-dependent risk pattern [18].

Alcohol-related HCC GWASs have widened this model. Trépo et al. conducted a case–control GWAS of alcohol-related HCC and identified common genetic variation associated with alcohol-related disease [13]. Buch et al. showed that genetic variation in *TERT* modifies HCC risk in alcohol-related cirrhosis, linking telomere regulation to inherited susceptibility in alcohol-associated hepatocarcinogenesis [14]. Large North American non-HBV studies have broadened the set of implicated loci. Hassan et al. identified high-impact susceptibility loci for HCC in North America, including genome-wide significant associations in non-viral or overall HCC in the *MOBP*, *TERT*, *TM6SF2*, *MAU2*, *PNPLA3*, *SAMM50*, and *PARVB* regions, and *HLA-DQB1* in HCV-positive HCC [15].

### 4.3. Meta-Analytic Consolidation

The field has shifted from focusing on discovering individual loci to adopting an integrative approach in genetic analysis. Ghouse et al. performed a large GWAS meta-analysis including 6540 HCC cases and 2,096,759 controls, followed by validation in two independent cohorts totalling 7630 cases and 733,689 controls. The study confirmed known associations in *PNPLA3*, *TM6SF2*, *TERT*, *IFNL4*, and *HLA-DP1* and identified genome-wide significant loci in *KLF15*, *HSD17B13*, *APOE*, *HFE*, and *MTARC1*. The inclusion of genetic correlation and Mendelian randomisation analyses provided further evidence of a link between obesity, hepatic steatosis, cirrhosis, and inherited HCC risk [16]. This meta-analysis marks a transition from small aetiology-specific GWASs towards larger cross-cohort discovery studies that can test shared and aetiology-specific effects.

Recent additions extend this trajectory. Li et al. identified HBV-related HCC susceptibility loci at *HLA-DQA1*/*HLA-DRB1* and *GRIK1* in chronic HBV carriers, expanding the early viral-HCC locus map beyond *KIF1B*, *STAT4*, *HLA-DQ*, *MICA*, and *DEPDC5* [23]. Chronic liver disease modifier studies have also become relevant to HCC interpretation because variants in *IFNL3*/*IFNL4*, *PNPLA3*, *TM6SF2*, *HSD17B13*, *GCKR*, *MBOAT7*, *APOE*, and *WNT3A*-*WNT9A* shape fibrosis, cirrhosis, alcohol-related HCC, MASLD-related HCC, or risk-model performance across high-risk cohorts [13,25,26,27,28]. These studies do not all constitute classical discovery GWASs for incident HCC. However, they provide useful GWAS-adjacent evidence, as HCC risk is mediated by chronic liver injury, cirrhosis, viral control, and metabolic traits.

**Figure 1:**
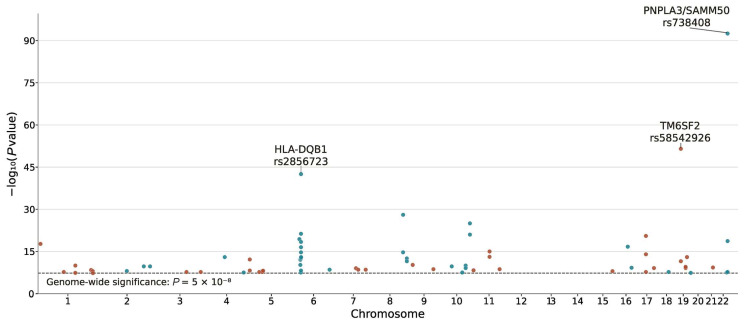
Manhattan plot of genome-wide significant HCC GWAS SNPs curated in the NHGRI-EBI GWAS Catalogue (trait ID: MONDO_007256). *N* = 72 unique SNPs with *P* ≤ 5 × 10^−8^ (dashed line). Only selected highly significant or manuscript-relevant loci are labelled to reduce clutter. Chromosome positions are GWAS Catalogue SNP mappings, and chromosome offsets use GRCh38 chromosome lengths for display. This plot summarises curated associations from multiple studies and should not be interpreted as the result of a single harmonised GWAS analysis.

To visualise the pattern of public associations, HCC-associated SNPs were retrieved from the NHGRI-EBI GWAS Catalogue’s HCC trait record and filtered for genome-wide significance [29]. The resulting Manhattan plot shows a dense immune-region signal on chromosome 6, a strong liver-metabolic signal around the *PNPLA3*/*SAMM50* region on chromosome 22, and additional high-significance loci, including *TM6SF2* on chromosome 19 and *TERT* on chromosome 5 (Figure 1). This distribution aligns with a recurring biological distinction in this review: viral HCC studies are enriched for immune and antigen-presentation loci, whereas non-viral and metabolic HCC studies implicate pathways related to lipid handling, telomeres, steatosis, and cirrhosis.

## 5. Fine-Mapping and Functional Annotation of HCC-Related GWAS Loci

Fine-mapping and functional annotation are required because GWASs usually identify associated regions rather than causal variants or genes. The most interpretable HCC loci are supported by expression, protein, co-localisation, or experimental data. In HBV-related HCC, Jiang et al. reported that the *STAT4* risk allele was associated with lower *STAT4* mRNA expression in tumour and adjacent non-tumour tissues, adding functional support to the statistical association [6]. In HCV-related HCC, the *MICA* rs2596542 risk allele was linked to lower soluble MICA levels, consistent with the hypothesis of impaired immune surveillance [7].

HLA-region signals require particularly careful annotation because the major histocompatibility complex exhibits complex linkage disequilibrium, high polymorphism, and ancestry-specific haplotype structure. HLA imputation, conditional analysis, and cross-ancestry replication are therefore necessary to distinguish causal alleles from regional tagging markers. Sawai et al. used HLA-focused analyses to refine HBV-related associations in the HLA class I region [12], while Lange et al. identified *HCP5* rs2244546 as a tagging SNP in the *MICA*/*HCP5* region for HCV-associated HCC [9].

Recent studies integrate GWASs with gene-prioritisation methods. Yu et al. reported a two-stage GWAS of HBV-related HCC and identified rs55718051 at the *FAM114A1* locus, with functional assays suggesting that the implicated variant regulates *FAM114A1* expression and that *FAM114A1* may have tumour-suppressive properties in liver cancer models [30]. Hassan et al. incorporated functional annotation, co-localisation, heritability estimation, and gene–gene interaction analyses into a North American HCC GWAS [15]. Zhang et al. conducted a TWAS of HCC susceptibility in East Asia and combined S-PrediXcan, S-MultiXcan, pathway enrichment, TCGA expression analysis, survival analysis, and DepMap dependency screening [19].

Despite these advances, TWASs, eQTL analysis, and Mendelian randomisation should be viewed as tools for prioritisation rather than conclusive proof of causality. TWASs rely on tissue specificity and matching ancestry in expression reference panels, while Mendelian randomisation can be influenced by horizontal pleiotropy and phenotypic heterogeneity. Robust causal inference will need agreement across fine-mapping, co-localisation, cell-type-specific epigenomics, perturbation experiments, and clinical phenotype data.

## 6. Application of GWAS Results

The primary uses of HCC GWAS findings include uncovering biological mechanisms, stratifying risk, and developing clinical models. At the discovery level, GWAS results help prioritise biological pathways for mechanistic research even when individual loci are not yet clinically actionable. These insights guide pathway selection for mechanistic studies, even when individual loci are not yet ready for clinical application.

Risk prediction is the most visible translational application, but it remains in its early stages. PRS studies are particularly relevant for MASLD and post-SVR populations, where traditional surveillance thresholds are less settled than in cirrhosis or chronic HBV. A study published under the historical NAFLD terminology in an East Asian population suggested that inherited liver-fat burden may help identify individuals at higher HCC risk [20]. Yu et al. also evaluated serum vitamin D and an HCC genetic risk score in the UK Biobank, combining genetic risk with a modifiable biomarker [21]. PRS implementation in HCC remains premature without prospective validation, ancestry-specific calibration, decision-curve analysis, cost-effectiveness evaluation, and demonstration of incremental benefit over established clinical variables, including cirrhosis, age, sex, platelet count, liver stiffness, viral status, alcohol exposure, diabetes, and body mass index.

GWAS results can also guide trial enrichment and surveillance strategies. Genotype-informed models might eventually pinpoint high-risk groups among patients with chronic HBV, post-SVR HCV, alcohol-related cirrhosis, or MASLD who could benefit from more frequent imaging or biomarker monitoring. Conversely, these models could help identify low-risk groups for whom surveillance intervals or methods might be adjusted. However, such uses need prospective validation, as a statistically significant risk allele does not always translate into better clinical decision-making.

## 7. Summary of Major Loci and Their Biological Functions

The representative loci in Table 1 support an aetiology-aware interpretation of HCC susceptibility, although immune, metabolic, fibrotic, and oncogenic pathways overlap within the chronically injured liver microenvironment.

**Figure 2:**
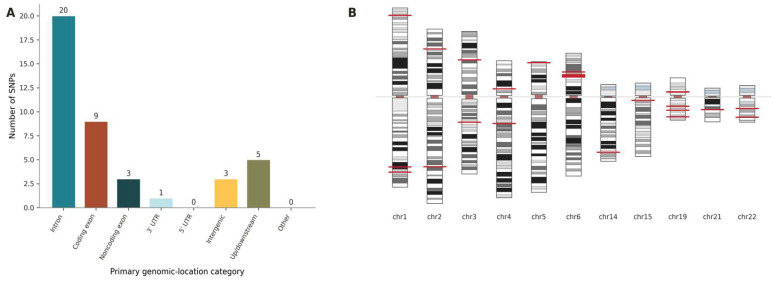
Genomic annotation and chromosomal distribution of the 41 representative SNPs listed in Table 1 after deduplication by GRCh38.p14 coordinates. (**A**) Primary genomic-location categories assigned using Ensembl VEP on the GRCh38.p14 primary assembly. (**B**) Ideograms for chromosomes containing Table 1 SNPs. Ideograms are aligned at the centromeres [32]. Red horizontal lines mark SNP genomic positions; chromosomes without Table 1 SNPs are not shown. Please note that SNPs have additional transcript-specific annotations (**A**).

To summarise the genomic context of the representative variants in Table 1, the 41 listed SNPs were annotated with the ENSEMBL Variant Effect Predictor (VEP) on the GRCh38.p14 primary assembly [31]. Since many variants have multiple transcript-specific effects, each SNP was assigned a single primary category based on the most interpretable VEP consequence class. Most representative HCC GWASs or GWAS-adjacent variants mapped to intronic or exonic regions, with additional annotations in intergenic, upstream/downstream, and 3′ UTR regions (Figure 2A). The SNP positions were also projected onto hg38 cytoband ideograms for the chromosomes listed in Table 1 (Figure 2B) [32]. This distribution is consistent with the broader observation that HCC GWAS signals often implicate regulatory or transcript-context-dependent mechanisms rather than simple protein-coding disruption.

## 8. Current Debates, Caveats, and Knowledge Gaps

### 8.1. Association Versus Causation

Many HCC GWAS signals remain statistical associations rather than established mechanisms. MICA, STAT4, FAM114A1, and some integrative meta-analysis loci are relatively strong because they have expression, protein, functional, or prioritisation support [6,7,16,30]. However, many variants are regional markers that require fine-mapping and experimental validation. Gene-name interpretation should therefore be cautious unless supported by convergent evidence from co-localisation, eQTL analysis, TWASs, biological perturbation, or other functional data.

### 8.2. Control Definition and Phenotype Resolution

Control selection remains a key challenge in the field. Healthy controls can enhance study power but may confound outcomes due to differences in the risks of infection, chronic liver disease, fibrosis, cirrhosis, and HCC. More clinically specific control groups—such as viral-carrier controls, cirrhosis controls, and post-SVR controls—are preferable but tend to be smaller and more difficult to recruit. Going forward, GWASs should differentiate among categories like chronic infection, advanced fibrosis, compensated cirrhosis, decompensated cirrhosis, post-SVR conditions, MASLD without cirrhosis, and established HCC.

### 8.3. Ancestry Representation and PRS Portability

GWAS research in HCC has developed through several overlapping phases. Early work was dominated by aetiology-specific discovery studies in chronic viral hepatitis, especially HBV-related HCC in Chinese cohorts and HCV-related HCC in Japanese cohorts, which identified susceptibility signals such as *KIF1B*/1p36.22, *MICA*, *DEPDC5*, *STAT4*, and *HLA-DQ* [5,6,7,8]. African populations warrant explicit consideration in future HCC GWAS and PRS studies. Sub-Saharan Africa carries a substantial burden of chronic HBV infection and HBV-associated HCC, and disease in this setting is often shaped by early-life infection, delayed diagnosis, limited access to antiviral treatment, and weak surveillance infrastructure [33,34]. Genetic studies in these populations are also particularly important because aflatoxin B1 exposure is a well-established hepatocarcinogenic cofactor in many African regions and can interact biologically with HBV-related carcinogenesis [35]. However, African and African-ancestry populations remain markedly underrepresented in human genomics, which limits locus discovery, fine-mapping, and PRS transferability for populations with high genetic diversity and distinct environmental exposures [36,37]. Expanding African HCC GWAS cohorts, with careful measurement of HBV status, HBV genotype, aflatoxin biomarkers, HIV or HDV coinfection, cirrhosis stage, and local ancestry, would improve the global relevance of the field and may identify ancestry- or exposure-specific susceptibility signals not captured in East Asian or European datasets [36,37].

A second phase addressed replication, ancestry transferability, and immune-region refinement, including HLA class I and *HCP5* analyses that clarified the role of antigen presentation and major histocompatibility complex variation in viral hepatocarcinogenesis [9,10,11,12]. More recent studies have extended the field towards alcohol-related, MASLD-related, post-SVR, and non-viral HCC, while incorporating meta-analysis, TWASs, colocalisation, Mendelian randomisation, PRS, and functional assays to move from locus discovery towards functional prioritisation, causal prioritisation, and clinical translation [13,14,15,16,17,19,20,21,30].

### 8.4. Clinical Utility

Currently, HCC surveillance decisions mainly rely on clinical factors like cirrhosis, chronic HBV status, age, sex, family history, and liver disease severity, rather than germline genotype. GWAS results are stronger as biological and risk-assessment evidence than as everyday clinical instruments. A genotype-based model must show better discrimination, calibration, net benefit, and cost-effectiveness before changing surveillance guidelines.

## 9. Cross-Disorder Risk Variants Between HCC and Other Diseases

Many HCC susceptibility loci are not HCC-specific. They are shared with upstream liver diseases, metabolic traits, viral response phenotypes, or other cancers. This cross-disorder architecture is biologically plausible because HCC usually arises from chronic liver injury, fibrosis, cirrhosis, immune dysregulation, or metabolic stress rather than from inherited cancer predisposition alone.

The *PNPLA3* rs738409 locus is the clearest example of a cross-disorder risk variant. It is associated with hepatic fat accumulation, steatohepatitis, fibrosis, cirrhosis, and HCC risk. Liu et al. showed that carriage of the *PNPLA3* rs738409 G allele was associated with increased risk of HCC in a cohort described using the historical NAFLD-related HCC terminology in European Caucasian patients, even after adjustment for age, sex, diabetes, body mass index, and cirrhosis status [38]. Yang et al. also found that *PNPLA3* rs738409 was associated with HCC across aetiologies, particularly alcohol-related liver disease [18]. This pattern suggests that *PNPLA3* influences HCC partly through progressive liver injury, but it may also modify carcinogenic risk within established liver disease.

*TM6SF2* rs58542926 is another cross-phenotype locus. It was first established as a susceptibility variant for steatotic liver disease in a study using the historical non-alcoholic fatty liver disease terminology through exome-wide association analysis [39], and subsequent HCC studies have implicated the same locus in alcohol-related, MASLD-related, and non-viral HCC contexts [15,18]. The biological interpretation is complex because *TM6SF2* variation can increase liver fat and liver disease risk while showing different directions of association with circulating lipid traits and cardiovascular phenotypes. Thus, *TM6SF2* illustrates how a variant can create organ-specific trade-offs between hepatic and cardiometabolic disease.

*HSD17B13* provides a particularly important example because loss-of-function variation appears to confer protection against chronic liver disease progression and HCC development. The protein-truncating splice variant rs72613567:TA was associated with a reduced risk of chronic liver disease and lower levels of markers of hepatocellular injury, supporting a biological model in which reduced *HSD17B13* activity may be protective rather than merely correlated with disease status [40]. Subsequent population-based analyses incorporating *PNPLA3*, *TM6SF2*, and *HSD17B13* further showed that the protective *HSD17B13* allele can partially offset the inherited risk conferred by steatogenic variants and is associated with a lower risk of cirrhosis and HCC [27]. A recent HCC GWAS meta-analysis also identified *HSD17B13* among loci associated with HCC risk, alongside *PNPLA3*, *TM6SF2*, *TERT*, *IFNL4*, *HLA-DP1*, *APOE*, *HFE*, and *MTARC1* [16]. Taken together, the findings of this analysis indicate that *HSD17B13* is one of the clearest examples in HCC genetics of a GWAS-supported locus that is reinforced by functional variant interpretation, liver disease biology, and clinically relevant protection against the upstream processes that drive hepatocarcinogenesis.

Cross-disorder susceptibility also extends beyond the canonical liver-fat loci to a wider metabolic and inflammatory genetic architecture. Variants used in hepatic fat-content polygenic scores, including *MBOAT7* and *GCKR* in addition to *PNPLA3* and *TM6SF2*, stratify risk of severe liver disease most strongly among individuals with obesity, diabetes, or fatty liver, suggesting that germline risk is modified by metabolic context [41]. Multi-ancestry cirrhosis genetics further supports this view, identifying loci enriched for hepatic lipid metabolism and showing that *PNPLA3* interacts with alcohol intake, obesity, and diabetes in relation to cirrhosis and HCC risk [42]. These observations are consistent with broader NAFLD/MASLD genetics, in which *GCKR*, *MBOAT7*, *PNPLA3*, and *TM6SF2* influence steatosis, fibrosis progression, and downstream cancer risk through lipid handling, insulin resistance, and fibrogenic pathways [43]. At the pathway level, HCC is also shaped by lipid metabolic reprogramming and chronic inflammatory signalling, including NF-κB, STAT3, stress-response, cytokine, and immune-regulatory networks that overlap with obesity, diabetes, and chronic liver injury [44,45]. Thus, cross-disorder HCC genetics should be interpreted as a convergence of inherited lipid-metabolic susceptibility, systemic metabolic traits, inflammatory signalling, and liver disease progression rather than as a small set of single-locus effects.

The cross-disorder architecture of HCC risk is not limited to the three liver-fat modifiers discussed above. A broader interpretation is supported by the recent HCC GWAS meta-analysis, in which risk loci also overlapped with hepatobiliary cancer, cirrhosis, hepatic steatosis, obesity-related traits, iron metabolism, and metabolic disease phenotypes [16]. *TERT* is a particularly informative example because it links HCC susceptibility to the wider biology of telomere maintenance, cellular ageing, tissue regeneration, and cancer predisposition. In alcohol-related cirrhosis, germline variation at *TERT* modifies HCC risk, while telomere-length GWASs and multi-cancer fine-mapping studies show that the same chromosomal region is relevant to leukocyte telomere length and risk of several malignancies, including glioma, lung cancer, melanoma, pancreatic cancer, and testicular cancer [14,46,47]. This suggests that the *TERT* signal in HCC should be interpreted as part of a wider telomere-maintenance axis rather than as a liver-specific association alone. *APOE* provides a second example of broader pleiotropy. The rs429358 locus has been associated with HCC risk among patients with cirrhosis and was also retained in the recent HCC meta-analysis, but *APOE* is best known for its effects on lipoprotein handling, Alzheimer’s disease, cardiovascular disease, and longevity [16,48,49]. Its inclusion among HCC-associated loci therefore connects hepatocarcinogenesis with systemic lipid transport, vascular-metabolic risk, ageing biology, and neurodegenerative disease genetics. Similar reasoning can be extended to *HFE* and *MTARC1*, which link HCC susceptibility to iron overload and mitochondrial redox/liver-injury pathways, respectively, and to interferon-lambda loci, which connect viral control, fibrosis progression, and immune-mediated disease biology. The cross-disorder section should therefore be framed not simply as a discussion of individual replicated liver loci, but as evidence that HCC susceptibility lies at the intersection of immune recognition, lipid metabolism, telomere biology, iron handling, ageing, and systemic metabolic disease. This broader framing also cautions against direct clinical translation of single-variant associations without phenotype-specific validation, because the same locus may have different or even opposing effects across liver disease, extrahepatic cancer, cardiovascular disease, and neurodegeneration.

## 10. Future Directions

Future research ought to focus on large, multi-ancestry consortia with standardised phenotype definitions and comprehensive exposure data. These studies should gather information on viral status, antiviral treatment history, SVR status, alcohol use, body mass index, diabetes, liver stiffness, platelet count, fibrosis stage, cirrhosis status, aflatoxin exposure if relevant, and family history. Such standardisation would help researchers differentiate between genetic factors influencing susceptibility to liver disease and those related to malignant transformation.

Functional follow-up should become standard for top loci. Fine-mapping, single-cell chromatin accessibility, liver-cell-type-specific eQTLs, CRISPR perturbation, organoid models, and integration with tumour and adjacent-tissue transcriptomics can help identify causal genes and pathways. TWASs and Mendelian randomisation serve as prioritisation tools rather than definitive proof, and researchers should triangulate their findings with experimental biology.

Prospective evaluation of clinical translation is essential. Future studies on PRS should investigate whether genetic risk adds predictive value for HCC beyond existing clinical models within cohorts with chronic HBV, post-SVR HCV, alcohol-related cirrhosis, and MASLD. Decision-curve analysis can help establish if genotype-guided surveillance offers net benefits. In the absence of such evidence, PRS should remain a research instrument and not influence adjustments to surveillance intensity.

## 11. Conclusions

GWAS research has established that inherited susceptibility contributes to HCC risk, but interpretation remains shaped by aetiology, ancestry, liver disease stage, and incomplete clinical translation. The next phase of research in this field should focus on multi-ancestry validation, disease-stage-aware controls, functional fine-mapping, and prospective clinical utility studies. The goal is to develop biologically grounded and clinically useful models that identify which patients with chronic liver disease are most likely to develop HCC and how surveillance or prevention might be individualised.

## Figures and Tables

**Table 1:** Representative HCC GWASs or GWAS-adjacent SNPs, risk alleles, genomic coordinates, and primary functional annotation.

SNP	Gene/Locus	Primary Biological Pathway	Position	Risk Allele	Genomic Location	Reference
rs17401966	*KIF1B*	Tumour suppression/apoptosis	chr1:10325413	A	intron	[5]
rs2642442	*MTARC1*	Mitochondrial redox metabolism/liver injury	chr1:220800221	T	intron	[16]
rs708113	*WNT3A-WNT9A*	Wnt signalling/liver regeneration	chr1:228005052	T	upstream gene	[13,28]
rs1260326	*GCKR*	Glucose and lipid metabolism	chr2:27508073	T	missense	[26]
rs7574865	*STAT4*	Immune signalling	chr2:191099907	G	intron	[6]
rs9842969	*MOBP*	Not yet established	chr3:39558686	C	intron	[15]
rs7628416	*KLF15*	Metabolic transcriptional regulation	chr3:126376169	C	downstream gene	[16]
rs55718051	*FAM114A1*	Tumour suppression/cell-growth regulation	chr4:38905096	C	intron	[30]
rs4089	*HSD17B13*	Hepatic lipid metabolism/hepatocyte injury	chr4:87300193	C	downstream gene	[16]
rs72613567	*HSD17B13*	Hepatic lipid metabolism/hepatocyte injury	chr4:87310242	TA	splice donor	[13,26,27,28]
rs10069690	*TERT*	Telomere maintenance	chr5:1279675	T	intron	[14,16]
rs2242652	*TERT*	Telomere maintenance	chr5:1279913	A	intron	[14,15,16]
rs144861591	*HFE*	Iron metabolism	chr6:26072764	T	intergenic	[16]
rs2523961	*HLA-I*	Antigen presentation	chr6:29971803	G	non-coding	[12]
rs3094137	*HLA-I*	Antigen presentation	chr6:30233096	G	intron	[12]
rs2596542	*MICA*	Antigen presentation/immune surveillance	chr6:31398818	A	non-coding	[7]
rs2244546	*HCP5*	MHC regulation/immune surveillance	chr6:31468056	C, G	non-coding	[9]
rs9272105	*HLA-DQA1/HLA-DRB1*	Antigen presentation	chr6:32632222	A	intron	[23]
rs9275224	*HLA-DQB1*	Antigen presentation	chr6:32692101	G	upstream gene	[15,16]
rs9275319	*HLA-DQ*	Antigen presentation	chr6:32698518	A	intergenic	[6]
rs3179778	*HLA-DPA1*	Antigen presentation	chr6:33068197	A	3’-UTR	[16]
rs28929474	*SERPINA1*	Protease inhibition/liver injury	chr14:94378610	T	missense	[16]
rs4778350	*PWRN4*	Non-coding RNA/gene regulation	chr15:23937326	A	intron	[17]
rs58542926	*TM6SF2*	Hepatic lipid metabolism	chr19:19268740	T	stop gained	[13,15,16,18,26,27]
rs187429064	*TM6SF2*	Hepatic lipid metabolism	chr19:19269704	G	missense	[28]
rs10401969	*SUGP1*	Lipid metabolism/RNA splicing	chr19:19296909	C	intron	[15]
rs58489806	*MAU2/TM6SF2*	Hepatic lipid metabolism	chr19:19346108	T	intron	[14,15]
rs3794991	*GATAD2A*	Chromatin regulation	chr19:19499787	T	intron	[15]
rs143988316	*TM6SF2*	Hepatic lipid metabolism	chr19:19556445	T	intergenic	[14]
rs12971396	*IFNL4*	Antiviral immune response	chr19:39247226	C	missense	[16]
rs12979860	*IFNL4/IFNL3*	Antiviral immune response	chr19:39248147	C	intron	[25]
rs8099917	*IFNL3/IFNL4*	Antiviral immune response	chr19:39252525	G	upstream gene	[25]
rs429358	*APOE*	Lipid transport/metabolism	chr19:44908684	T	missense	[16,28]
rs641738	*MBOAT7*	Phospholipid remodelling	chr19:54173068	T	missense	[16,26,28]
rs455804	*GRIK1*	Glutamate signalling	chr21:29773850	C	intron	[23]
rs1012068	*DEPDC5*	Cell growth/mTOR-related signalling	chr22:31869917	G	intron	[8]
rs738409	*PNPLA3*	Hepatic lipid metabolism	chr22:43928847	G	missense	[13,15,18,26,27]
rs738408	*PNPLA3*	Hepatic lipid metabolism	chr22:43928850	T	missense	[16]
rs2294915	*PNPLA3*	Hepatic lipid metabolism	chr22:43945024	C	intron	[14]
rs2294922	*SAMM50*	Mitochondrial metabolism/lipid handling	chr22:43983685	C	intron	[15]
rs2401514	*PARVB*	Cell adhesion/extracellular-matrix signalling	chr22:43998139	A	intron	[15]

## Data Availability

No new data were created or analysed in this study. Data sharing does not apply to this article.

## Supplementary Materials

The following supporting information can be downloaded at: https://www.mdpi.com/article/10.3390/genes17070759/s1, Supplementary File S1: LLM-readable hand-off document describing the methodology of HCC GWAS expanded SNP source-literature search.

## Abbreviations

The following abbreviations are used in this manuscript:

eQTL	Expression quantitative trait locus
GWAS	Genome-wide association study
HBV	Hepatitis B virus
HCC	Hepatocellular carcinoma
HCV	Hepatitis C virus
MASLD	Metabolic dysfunction-associated steatotic liver disease
MASH	Metabolic dysfunction-associated steatohepatitis
MHC	Major histocompatibility complex
NAFLD	Non-alcoholic fatty liver disease; historical terminology, now broadly replaced by MASLD
NASH	Non-alcoholic steatohepatitis; historical terminology, now broadly replaced by MASH
OR	Odds ratio
PRS	Polygenic risk score
SLD	Steatotic liver disease
SNP	Single-nucleotide polymorphism
SVR	Sustained virologic response
TWAS	Transcriptome-wide association study
